# The Stl repressor from *Staphylococcus aureus* is an efficient inhibitor of the eukaryotic fruitfly dUTPase

**DOI:** 10.1002/2211-5463.12302

**Published:** 2017-12-27

**Authors:** András Benedek, István Pölöskei, Olivér Ozohanics, Károly Vékey, Beáta G. Vértessy

**Affiliations:** ^1^ Institute of Enzymology Research Centre for Natural Sciences Hungarian Academy of Sciences Budapest Hungary; ^2^ Department of Applied Biotechnology Budapest University of Technology and Economics Hungary; ^3^ Institute of Organic Chemistry Research Centre for Natural Sciences Hungarian Academy of Sciences Budapest Hungary

**Keywords:** dUTPase, inhibition, Stl

## Abstract

DNA metabolism and repair is vital for the maintenance of genome integrity. Specific proteinaceous inhibitors of key factors in this process have high potential for deciphering pathways of DNA metabolism and repair. The dUTPase enzyme family is responsible for guarding against erroneous uracil incorporation into DNA. Here, we investigate whether the staphylococcal Stl repressor may interact with not only bacterial but also eukaryotic dUTPase. We provide experimental evidence for the formation of a strong complex between Stl and *Drosophila melanogaster* dUTPase. We also find that dUTPase activity is strongly diminished in this complex. Our results suggest that the dUTPase protein sequences involved in binding to Stl are at least partially conserved through evolution from bacteria to eukaryotes.

**Abbreviations**
DSF: differential scanning fluorimetry
EMSA: electrophoretic mobility shift assay
GST: glutathione *S*‐transferase
SaPIs: *Staphylococcus aureus* pathogenicity islands

DNA integrity and the fidelity of DNA replication are of vital importance. The dUTPase enzyme family, ubiquitous in free‐living organisms 1 with some exceptions 2, contributes to these key issues by regulating the cellular dUTP/dTTP ratio. dUTPases catalyze the pyrophosphorolysis of dUTP into dUMP and pyrophosphate, providing the dUMP precursor for thymidylate biosynthesis. This enzymatic reaction also promotes clearance of dUTP from the cellular milieu, thereby preventing DNA polymerases from introducing dUMP moieties into DNA 1. The significance of this sanitizing action is due to the fact that most DNA polymerases cannot distinguish between dUTP and dTTP and will readily utilize either of these two building blocks, depending only on their relative availability 3, 4.

Elimination or inhibition of dUTPase activity leads to massive uracil incorporation into DNA that provokes futile hyperactivation of the base‐excision repair pathway and results in DNA strand breaks followed by chromosome fragmentation and cell death 5, 6. This cell death pathway is usually referred to as ‘thymine‐less cell death’ and may also be induced by chemotherapeutic drugs interfering with *de novo* thymidylate biosynthesis, such as fluoropyrimidines and methotrexate derivatives 7. In fact, this chemotherapeutic strategy is frequently used clinically both against neoplastic diseases and against pathogenic microorganisms 3, 7, 8, 9, 10. Inhibition of dUTPase by small molecular drugs may also enhance the effectivity of this clinical protocol 11. Several small molecular dUTPase inhibitors have been identified in the literature 12, 13, 14, 15. A proteinaceous dUTPase inhibitor, namely the staphylococcal Stl repressor, has also been discovered recently and it was shown to be active against trimeric dUTPases of several staphylococcal phages, as well as against the trimeric mycobacterial dUTPase 16, 17, 18.

Most probably, this interesting cross‐species effect needs to be necessarily associated with structural features present in both phage and mycobacterial dUTPases. Notably, as most dUTPases belong to the all‐β dUTPase enzyme family, the main structural fold is well preserved not just among prokaryotic dUTPases, but also in eukaryotic ones 19, 20. Within the evolutionary conserved dUTPase fold, three β‐pleated polypeptide subunits form a trimeric enzyme possessing three equivalent active sites situated at the intersubunit clefts 21, 22. Although the overall conservation of the fold is clearly a major characteristic of the all‐β dUTPase enzyme family, at the residue level only those residues are conserved that are directly involved in active site architecture 20, 23. Other protein surfaces potentially available for binding a macromolecular partner show great variation with respect to polarity, charge distribution, H‐bonding, and Van der Waals capabilities. Therefore, it is an intriguing question to investigate whether any eukaryotic dUTPase may also form a protein–protein complex with the staphylococcal Stl. It is worthwhile to note that in the case of the enzyme family of uracil‐DNA glycosylases, the UGI inhibitor protein (from the *Bacillus subtilis* phage PBS2) is fully functional in complexation and inhibition of not only prokaryotic, but also human and other eukaryotic uracil‐DNA glycosylases, presenting a potentially relevant parallel situation 24, 25, 26.

In the dUTPase–Stl interaction investigated so far, functional effects of the complexation result not only in enzymatic inhibition of dUTPase, but also in perturbation of the repressor function of Stl 16, 17, 27. In *Staphylococcus aureus*, Stl is responsible for repressing replication of SaPIbov1 pathogenicity island (SaPI) 28. *Staphylococcus aureus* pathogenicity islands (SaPIs) are mobile genetic elements being responsible for horizontal gene transfer, a process being important for bacterial evolution 29, 30. Transcription of the SaPI may be induced upon helper phage infection by a specific interaction partner, which in the case of SaPIbov1 Stl is the helper phage dUTPase 27. It was also shown that dUTPase removes Stl from its bound DNA 16, 17, 18.

In the present study, we wished to investigate whether Stl is able to form a stable complex with the eukaryotic *Drosophila melanogaster* dUTPase *in vitro*. Further on, we decided to determine the functional effects following from this complexation. Our results obtained by several independent methodologies show that a strong complex is formed between Stl and *Drosophila* dUTPase, similar to the case with phage and mycobacterial dUTPases. In this complex, dUTPase enzymatic activity is significantly reduced, but DNA binding to Stl may still be possible.

## Materials and methods

### Protein expression and purification

The Stl‐encoding gene sequence has been inserted into pGEX‐4T‐1 vector allowing glutathione *S*‐transferase (GST) fusion expression and purification. The *D. melanogaster* dUTPase gene has been ligated into pET‐15b vector between the *Bam*HI and *Nde*I cleavage sites resulting in translation of a His‐tagged dUTPase construct enabling purification with Ni‐NTA affinity chromatography. Both constructs were expressed in *Escherichia coli* BL21 (DE3) Rosetta cells under similar conditions (cf. also 17, 31, 32).

For protein expression, 0.5 L of LB medium was inoculated with a 5 mL overnight cell culture and grown at 37 °C until OD_600_ reached 0.5. At this point, protein expression was induced by the addition of 0.5 mm isopropyl β‐d‐1‐thiogalactopyranoside. The cell cultures were incubated for further 4 h at 37 °C for *D. melanogaster* dUTPase and 30 °C for protein Stl. After centrifugation at 1376 ***g*** for 20 min at 4 °C, cell pellets were resuspended in 15 mL precooled PBS and centrifuged again at 1376 ***g*** for 20 min at 4 °C, then stored at −80 °C until further usage.

Cells containing *D. melanogaster* dUTPase were resuspended in 50 mL of 50 mm TRIS/HCl solution containing 300 mm NaCl, 0.5 mm EDTA, 0.1% Triton‐X 100, 1 mm PMSF, 5 mm benzamidine, EDTA‐free protease inhibitor cocktail tablet, 10 mm β‐mercaptoethanol, 0.1 mg·mL^−1^ lysozyme, 0.1 mg·mL^−1^ DNase, and 0.01 mg·mL^−1^ RNase A at pH 8.0. The suspension was sonicated, centrifuged, then applied onto a benchtop nickel/nitriloacetic acid/agarose affinity chromatography column. The protein was eluted with 50 mm HEPES containing 30 mm KCl, 500 mm imidazole, 10 mm β‐mercaptoethanol, 0.1 mm PMSF, and EDTA‐free protease inhibitor cocktail tablet at pH 7.5. The eluted samples were dialyzed overnight into 20 mm HEPES, 100 mm NaCl, 5 mm MgCl_2_, and 10 mm β‐mercaptoethanol at pH = 7.5 (dUTPase buffer). The sample was concentrated and further purified on a Superose 12 10/300 GL column (GE Healthcare, Little Chalfont, UK). Final sample concentration was carried out using an ultrafiltration membrane (Amicon Ultra‐4, Merck‐Millipore, Darmstadt, Germany).

Cells containing protein Stl in a GST‐fused form were resuspended in 30 mL of 50 mm TRIS/HCl containing 1 m NaCl, EDTA‐free protease inhibitor cocktail tablet, 10 mm dithiothreitol, 0.1 mg·mL^−1^ DNase, and 0.01 mg·mL^−1^ RNase A at pH 7.5. After sonication and centrifugation, the suspension was applied onto a glutathione column. Stl elution was carried out by cleavage of the column‐bound GST tag using 80 units of thrombin in 3 mL reaction volume.

Protein concentrations were measured spectrophotometrically (Nanodrop 2000c, Thermo Scientific) from 280‐nm absorbance values.

### Size exclusion chromatography (SEC)

Size exclusion chromatography (SEC) was carried out on AKTA FPLC purification system using a Superose 12 10/300 GL column (GE Healthcare) previously equilibrated with dUTPase buffer (20 mm HEPES, 100 mm NaCl, 5 mm MgCl_2_, 10 mm β‐mercaptoethanol, pH = 7.5). Complex formation was estimated based on comparison of the peak elution volumes of separate proteins with the value corresponding to the mixture of the two components. Fractions of 0.5 mL were collected after each injection. Peak elution fractions were concentrated on Amicon Ultra‐4 ultrafiltration membranes (Merck‐Millipore) before subsequent mass spectrometric analysis.

### Differential scanning fluorimetry (DSF)

Samples were heated from 20 to 80 °C using three parallels of each measurement. For visualization of protein unfolding, Sypro Orange protein dye was used in 1000‐fold dilution. Melting points were obtained as global minimums corresponding to the first derivate of the melting curve.

### Mass spectrometry

Mass spectra were measured in positive ion mode using a Waters QTOF Premier instrument with electrospray ionization source. Native conditions were applied; that is, ions were generated from aqueous 5 mm NH_4_HCO_3_ buffer solution (pH: 7.8) containing the protein at 1 μm monomer concentration. Under such conditions, native protein complexes can be transferred from the solution to the gas phase. The capillary voltage was 2800 V, the sampling cone voltage was 128 V, and the temperature of the source was kept at 90 °C. Mass spectra were recorded in the mass range of 1500–6000 *m*/*z*.

### Enzyme activity and inhibition assay

These measurements were taken according to our previously used protocol 17. Hydrolysis of dUTP results in proton release to the solution which can be followed as a change in pH. To quantify this, phenol red indicator was added to the reaction mixture (1 mm HEPES, 150 mm KCl, 5 mm MgCl_2_, 40 μm phenol red, pH: 7.5) and its absorbance was measured continuously as a function of time at 559 nm and 293 K using a 10‐mm path length plastic cuvette. Initial velocity was determined from the first 10% of the progress curve. At least three parallel measurements were taken in all cases.

For Stl inhibition measurements, 100 nm of dUTPase and different amounts of protein Stl were preincubated together for 5 min in the measuring buffer at 293 K. The enzymatic reaction was always initiated by the addition of the dUTP molecule after mixing all other components.

### Electrophoretic mobility shift assay (EMSA)

Electrophoretic mobility shift assay (EMSA) experiments were carried out on 8% TRIS/borate/EDTA (TBE) gels using a double‐stranded 43‐mer oligonucleotide (corresponding to the oligo termed ‘Inter‐R’ in 33, with the sequence ‘tcctcgaacaaattatctcacatcgagatatttatttcaacat’ representing the Stl‐specific DNA binding site. Samples were mixed in ‘EMSA buffer’ (TBE pH = 7.5, 100 mm NaCl, 0.5 mm EDTA). Before loading onto the gel, samples were incubated for 15 min at 293 K. After 1‐h pre‐electrophoresis of the empty gel on 150 V, the samples were run using the same voltage for 45 min at room temperature. GelRed was used to stain DNA. DNA bands were visualized by UVI‐Tec gel documentation system after 15 min.

### Native gel electrophoresis

Native gel electrophoresis was set up in a two‐phase polyacrylamide gel. Acrylamide concentration was 4% in the stacking gel (pH = 6.8) and 10% in the resolving gel (pH = 8.8). After 30‐min pre‐electrophoresis without sample addition at constant 100 V on ice, the electrophoresis was performed for another 2.25 h at 200 V in native ‘ELFO buffer’ (30.3 g·L^−1^ TRIS base, 144 g·L^−1^ glycine, pH = 8.7). During electrophoresis, the whole apparatus was placed on ice. The gel was stained with Coomassie Brilliant Blue G250 dye (Thermo Fisher Scientific, Waltham, MA, USA).

### Structural and homology modeling

Three‐dimensional structural views were created by pymol (version 0.99rc6) 34. The Clustal Omega server was used for multiple sequence alignment 35. *Drosophila melanogaster* dUTPase structure was visualized as a homology model based on the human dUTPase crystal structure (PDB 3EHW) using the SWISS‐MODEL server 36.

## Results and Discussion

### Staphylococcal Stl forms a stable complex with *Drosophila* dUTPase

**Figure 1:**
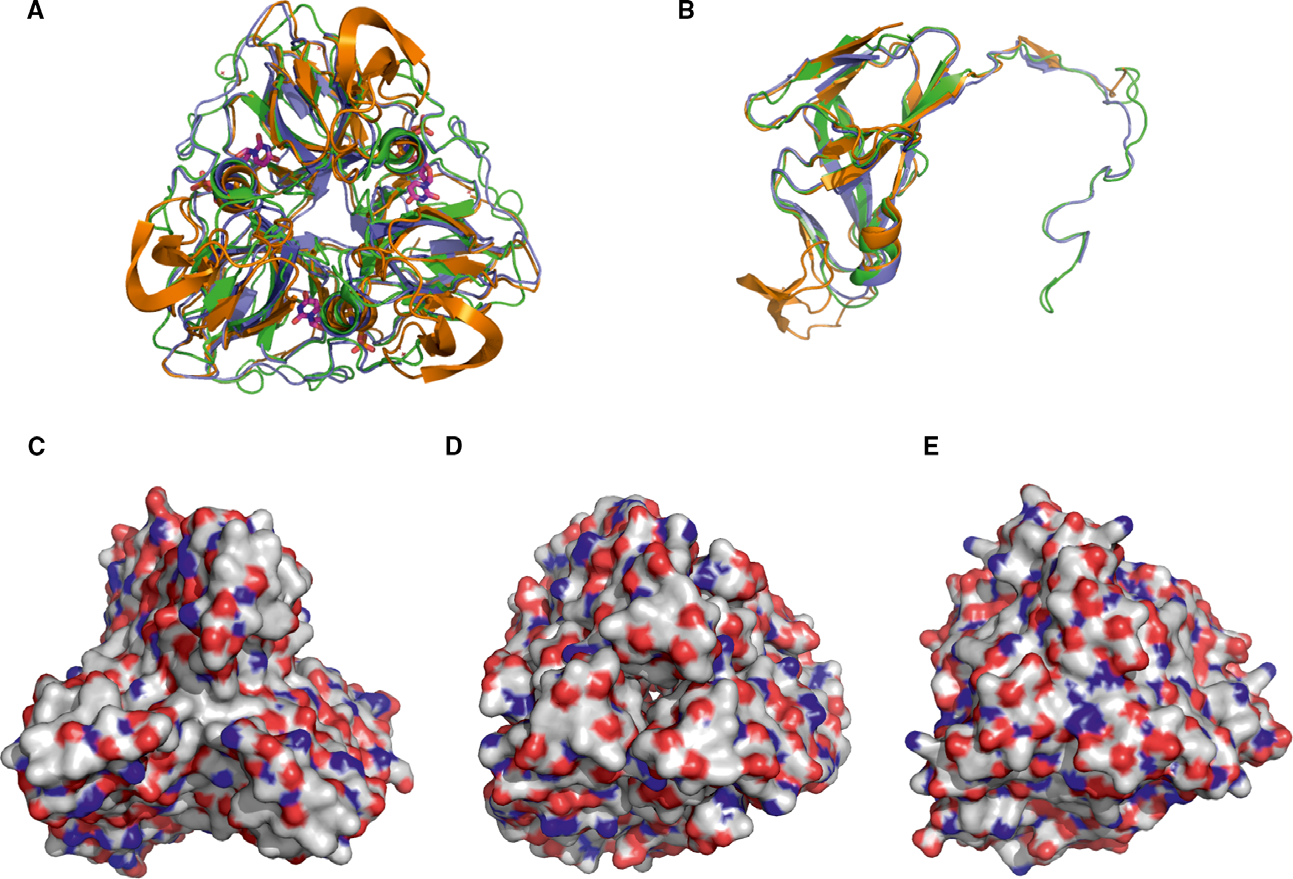
Three‐dimensional structural comparison of three dUTPases interacting with Stl. (A,B) Trimeric and monomeric overlay of 3D crystal structures. Orange color code stands for Φ11 phage dUTPase (PDB 4GV8), green for *M. tuberculosis* (PDB 2PY4), and blue for *Drosophila melanogaster* (built using PDB 3EHW) dUTPase. (C,D,E) Surface representation of the Φ11 phage, *M. tuberculosis* and *D. melanogaster* dUTPase 3D structures, respectively. Color code: gray for carbon, blue for nitrogen, and red for oxygen atoms.

Figure 1 presents a structural alignment of one phage (*S. aureus* Φ11), one prokaryotic (*Mycobacterium tuberculosis*), and one eukaryotic (*D. melanogaster*) dUTPase 37, 38. It is clearly shown that the overall fold is well conserved both at the subunit and at the functional homotrimer level (Fig. 1A,B). However, surface representation shown at the same orientation for the three trimeric dUTPases (Fig. 1C,D,E) presents largely varied distribution of polar, charged, and hydrophobic surfaces. Despite this variation, the interaction among the mycobacterial dUTPase and Stl shows similar characteristics to the staphylococcal phage dUTPase–Stl interaction 16.

**Figure 2:**
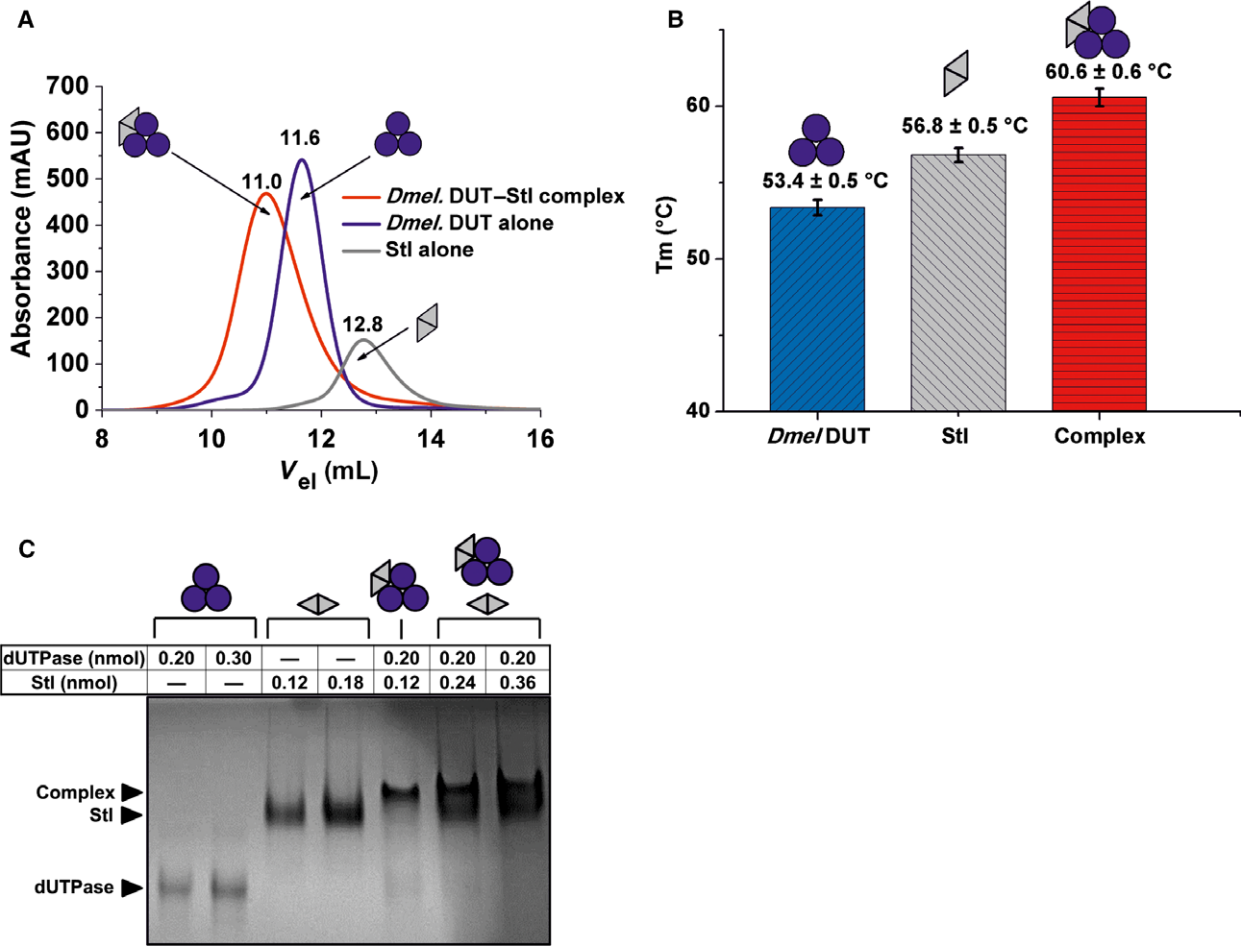
Complex formation between *Drosophila melanogaster* dUTPase and Stl. Color code: Blue circles and gray triangles indicate *D. melanogaster* dUTPase monomers and Stl monomers, respectively. Pictograms indicate their respective oligomer assembly. (A) Size exclusion chromatography. Blue line stands for dUTPase, gray line for Stl, and red line for their complex elution peak. Numbers above the lines show exact peak elution volumes for better comparison. (B) DSF. Bar graph shows melting points of the separate proteins and their complex. Means and standard deviations are indicated on the graph. (C) Native polyacrylamide gel electrophoresis. Names with black arrows on the left of the gel identify the different protein bands. The table on the top shows the molar amounts of proteins. Two separately drawn pictograms stand for two distinct protein bands within one lane.

To study the potential binding of fruitfly dUTPase to Stl, first we applied size exclusion chromatography as a widely used straightforward technique to investigate protein–protein interactions. The chromatograms shown in Fig. 2A clearly indicated that a complex is formed in the mixture of the two protein components and this complex elutes at a position associated with higher molecular mass as compared to either of the other two components. The size exclusion chromatography experiment also allowed us to conclude that the complex of the two proteins is stable enough to be withheld in the complex state upon the dilution that necessarily occurs during the gel filtration process.

To check whether the presumed complex formation has any effect on protein stability, another well‐characterized technique was used. Namely, we have performed differential scanning fluorimetry (DSF) experiments and found that indeed, the complexation induced a higher thermal stability, as the estimated melting temperature of 53.3 °C for dUTPase and 57.3 °C for Stl was shifted to 60.3 °C in the mixture of the two proteins (Fig 2B).

While these two independent experiments provided unequivocal evidence for the physical contact between the two proteins (Stl from the prokaryote *S. aureus* and dUTPase from the eukaryote *D. melanogaster*), these data did not allow an estimation of the stoichiometry of the complex. To continue our investigations, we therefore initiated native gel experiments as in our earlier studies 39. Data shown in Fig. 2C argue that on the one hand, the complex between staphylococcal Stl and *D. melanogaster* dUTPase is clearly visible on the native gel, while on the other hand, using different stoichiometric mixtures, bands corresponding to the separate components almost completely disappear at one exact component ratio being close to 3 : 2 dUTPase–Stl monomer assembly. Interestingly, this 3 : 2 stoichiometry was also suggested in the complex of the staphylococcal Φ11 phage dUTPase and Stl 17.

**Figure 3:**
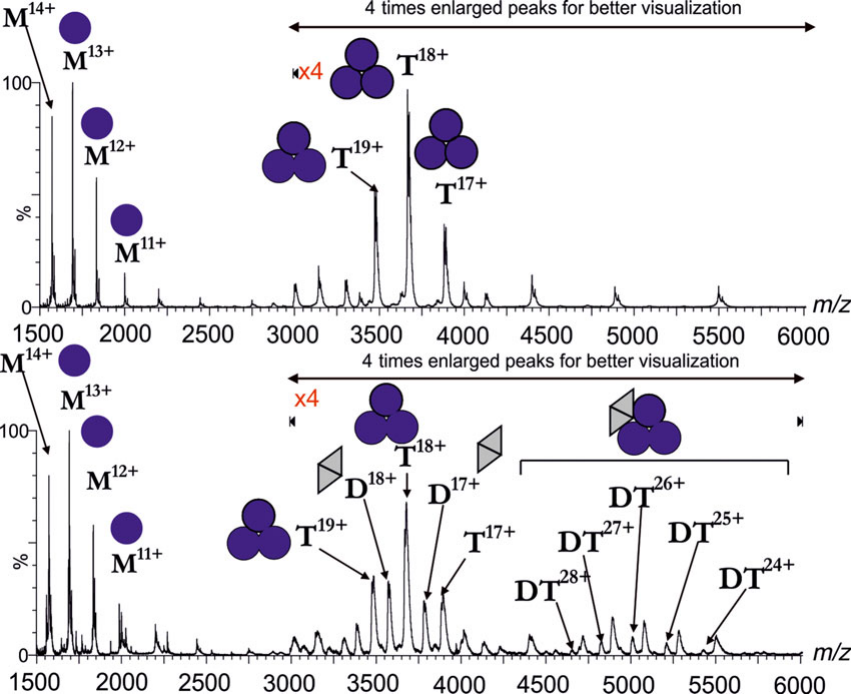
Mass spectrometric analysis of the gel‐filtrated *Drosophila melanogaster* dUTPase (upper panel) and dUTPase–Stl complex (lower panel). Blue circles symbolize *D. melanogaster* dUTPase monomers; gray triangles denote Stl monomers. Letters M, D, T, and DT denote monomeric, dimeric, trimeric, and complex molecular ion peaks, respectively, while numbers denote the charge states. Upper panel: *D. melanogaster* dUTPase alone; lower panel: complex of *D. melanogaster* dUTPase and Stl. For this measurement, peak elution fractions of the previous gel filtration were used.

To further study whether this stoichiometry may be valid, we decided to analyze the gel‐filtrated complex by mass spectrometry. Our aim was to compare the molecular ionic species present in the mixture of the two proteins to those molecular ions that are present in the separate solutions of the two proteins. Figure 3 upper panel shows the mass spectrum of the fruitfly dUTPase on its own. Mass data indicate that the trimeric fruitfly dUTPase can dissociate into monomers under the mass spectrometric conditions. This phenomenon was also observed in the case of a specific construct of *Drosophila virilis* dUTPase 40. Mass spectra for the Stl protein on its own were already published 17 and showed the presence of both monomeric and dimeric Stl species under the experimental condition of native mass spectrometry.

Mass spectra of the gel‐filtrated complex of Stl and dUTPase—shown on the lower panel of Fig. 3—present unequivocal evidence for the complexation of these two proteins. Namely, a species with a molecular mass corresponding to the complex formed between one dUTPase trimer and one Stl dimer (or two Stl monomers) is observed. This stoichiometry is also in agreement with the data of the native gel experiment.

### Functional effects of complex formation between Stl and *Drosophila melanogaster* dUTPase

Having found that fruitfly dUTPase and the staphylococcal Stl protein form a stable complex that resists experimental conditions of gel filtration, native gel electrophoresis, and mass spectrometry, we wished to determine the putative functional effects of this complexation. First, we tested whether Stl may inhibit the enzymatic activity of fruitfly dUTPase, as shown previously for phage and mycobacterial dUTPases.

**Figure 4:**
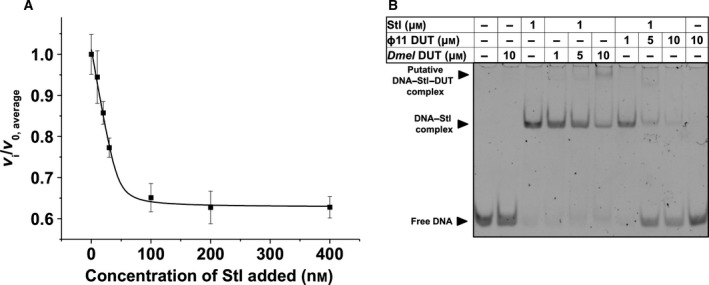
Functional effects of complex formation between *Drosophila melanogaster* dUTPase and Stl. (A) Protein Stl inhibits enzymatic activity of *D. melanogaster* dUTPase. Panel shows average and standard deviation of three parallel measurements. A quadratic binding equation was fitted to the data (solid black curve), from which the IC_50_ = 30 ± 5 nm value was calculated. (B) EMSA experiment shows that the *D. melanogaster* dUTPase–Stl interaction does not disrupt DNA–Stl complexation, differently from the Φ11 dUTPase–Stl interaction. Complexation leads to an upward shift in DNA positions, while complex disruption results in free DNA reappearance in its lower position. Please note that only DNA bands are visible on this gel.

As shown in Fig. 4A, there is a dose‐dependent activity loss of dUTPase upon the addition of increasing concentrations of Stl to the reaction mixtures. In these experiments, the two proteins were preincubated before the addition of the dUTP substrate to start the enzymatic reaction, as previously in the case of phage and mycobacterial dUTPases 16, 17.

The apparent IC_50_ of Stl determined in these enzyme inhibition experiments was calculated to be 30 ± 5 nm. This value is in good agreement with the IC_50_ of Stl determined for phage and mycobacterial dUTPase. However, the total inhibition observed at saturating Stl concentrations was ~ 40%, to be compared with almost 100% inhibition for Φ11 phage dUTPase and 80% inhibition for the mycobacterial dUTPase 16, 17. These characteristics indicate that although the complexation proceeds similarly, the actual inhibitory capacity of Stl within the different complexes reflects some species‐specific differences.

We also studied whether fruitfly dUTPase may perturb Stl–DNA complexation. In the EMSA experiment presented in Fig. 4B, we could nicely reproduce the previously published effect of Φ11 dUTPase on the DNA–Stl complex. Namely, the gel lanes show that increasing amount of Φ11 dUTPase leads to reappearance of the DNA band associated with the free DNA form (not bound to protein). However, when fruitfly dUTPase was added to the DNA–Stl complex, we could not observe dissociation of DNA from the DNA–Stl complex. Instead, a new band has appeared at a higher position on the gel which we putatively identified as a ternary complex (DNA–Stl–dUTPase). Although the existence of this putative ternary complex needs further investigation, it is evident based on the presented data that the fruitfly dUTPase does not necessarily disrupt the interaction between Stl and its cognate DNA sequence.

### Possible structural background of species‐specific differences in Stl‐induced dUTPase inhibition

**Figure 5:**
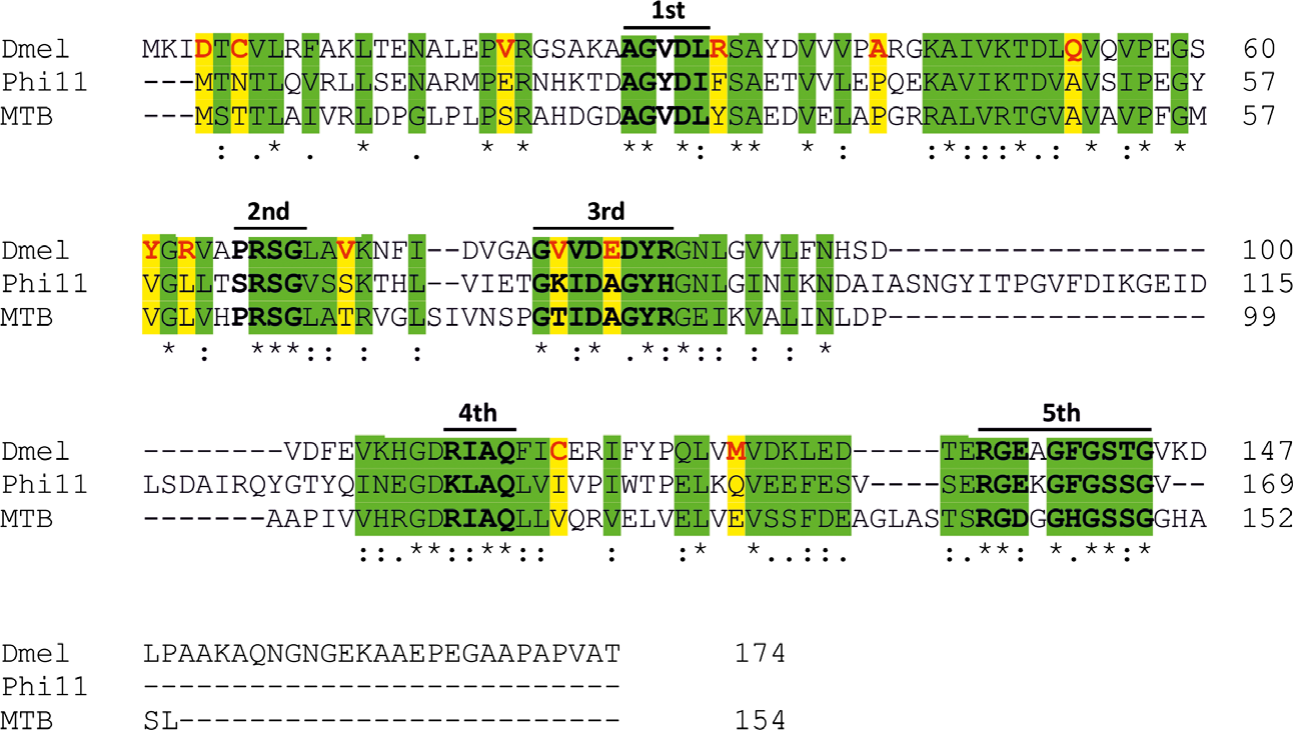
Species‐specific sequence similarities and alterations among three Stl‐inhibited all‐β dUTPases. Conserved sequence motifs are highlighted with bold black case and upper lines. Amino acids with similar side‐chain characteristics are shown in green background. Residues that are similar to Φ11 and *M. tuberculosis* dUTPases but different in the *Drosophila melanogaster* enzyme have yellow background. *Drosophila melanogaster* dUTPase residues with alternate characteristics are emphasized with red letters.

While all three dUTPases mentioned in this study can be inhibited by Stl *in vitro*, a remarkable difference is present in the maximal degree of their inhibition. To provide a putative explanation for these differences, we have aligned their sequences (Fig. 5). We have highlighted all amino acids possessing the same or at least similar side‐chain characteristics—these segments may be accordingly involved in Stl binding (cf. also 18). We were also looking for amino acid positions which are identical or similar in Φ11 and *M. tuberculosis* dUTPases but are of different characteristics in *D. melanogaster* dUTPase. Such residue alterations could serve as a basis for the observed significant difference in the degree of inhibition by Stl. The *D. melanogaster* dUTPase contains a *Drosophila* specific C‐terminal extension, which may alter steric properties or surface charge characteristics of the enzyme. The Φ11 dUTPase has a phage‐specific insert between the third and fourth sequence motifs 18, and the *M. tuberculosis* enzyme has a short mycobacteria‐specific surface loop just before the fifth motif 41. These factors may also be important for the differences in dUTPase interaction with protein Stl.

## Conclusions

Protein–protein interactions can be conserved among species, especially if orthologue components of a given complex are present in the different species. In the present work, our focus was somewhat different: We investigated whether a eukaryotic representative of the evolutionary conserved dUTPase enzyme family may bind to a staphylococcal repressor (Stl) that is not present in other species. We found that *D. melanogaster* dUTPase and Stl form a strong complex with significant functional effect on dUTPase enzymatic activity, parallel to Stl‐induced inhibition of phage and prokaryotic dUTPases. We conclude that Stl may be considered as a useful tool for specific inhibition of dUTPases in diverse systems. Further studies in progress in our laboratory will reveal whether *in vivo* dUTPase inhibition can be achieved in *D. melanogaster* model organism transfected with Stl.

## Author contributions

Planning the experiments: AB, OO, KV, and BGV. Performing the experiments: AB, IP, and OO. Analyzing data: AB, OO, and IP. Writing the paper: AB and BGV.
